# Development of a constitutive and an auto-inducible high-yield expression system for recombinant protein production in the microalga *Nannochloropsis oceanica*

**DOI:** 10.1007/s00253-020-10789-4

**Published:** 2020-09-09

**Authors:** Imke de Grahl, Sweta Suman Rout, Jodi Maple-Grødem, Sigrun Reumann

**Affiliations:** 1 Plant Biochemistry and Infection Biology, Institute of Plant Science and Microbiology, Universität Hamburg, Ohnhorststr. 18, D-22609 Hamburg, Germany; 2 The Norwegian Centre for Movement Disorders, Stavanger University Hospital, N-4021 Stavanger, Norway; 3 Department of Chemistry, Bioscience and Environmental Engineering, University of Stavanger, N-4036 Stavanger, Norway

**Keywords:** *Nannochloropsis oceanica*, Recombinant protein production, Constitutive and inducible promoters, Flow cytometry, Auto-induction medium, Venus

## Abstract

**Abstract:**

Photoautotrophic microalgae offer a great potential as novel hosts for efficient recombinant protein production. *Nannochloropsis oceanica* produces an extraordinarily high content of polyunsaturated fatty acids*,* and its robust growth characteristics, published genome sequence and efficient nuclear transformation make *N. oceanica* a promising candidate for biotechnological applications. To establish a robust and flexible system for recombinant protein production, we cloned six endogenous, potentially constitutive or inducible promoters from *N. oceanica* strain CCMP1779 and investigated their strength using monomeric *Venus* as reporter gene. Microscopic pre-screening of individual transformants revealed that the promoters of elongation factor (EF), tubulin (TUB) and nitrate reductase (NR) enabled high reporter gene expression. Comparative quantitative analyses of transformant populations by flow cytometry and qRT-PCR demonstrated the highest *Venus* expression from the EF promoter and the NR promoter if extended by an N-terminal 14-amino acid leader sequence. The kinetics of reporter gene expression were analysed during photobioreactor cultivation, achieving Venus yields of 0.3% (for EF) and 4.9% (for NR::LS) of total soluble protein. Since inducible expression systems enable the production of toxic proteins, we developed an auto-induction medium for the NR promoter transformants. By switching the N source from ammonium to nitrate in the presence of low ammonium concentrations, the starting point of Venus induction could be fine-tuned and shifted towards exponential growth phase while maintaining high recombinant protein yields. Taken together, we demonstrate that a model recombinant protein can be produced robustly and at very high levels in *N. oceanica* not only under constitutive but also under auto-inducible cultivation conditions.

**Key points:**

*• Nannochloropsis oceanica might serve as host for recombinant protein production.*

*• Comparative promoter strength analyses were conducted for twelve different constructs.*

*• Robust high-yield recombinant protein production was achieved under constitutive conditions.*

*• The nitrate reductase promoter enabled protein production under auto-induction conditions.*

**Electronic supplementary material:**

The online version of this article (10.1007/s00253-020-10789-4) contains supplementary material, which is available to authorized users.

## Introduction

Microalgae are photosynthetic unicellular organisms that are highly diverse in size, shape and internal cell structures (Andersen 2013). As primary producers in the aquatic environment, their immense CO_2_ assimilation capacity has a major impact not only on the global climate and the carbon cycle but also accounts for immense biomass production (Stephenson et al. 2011). Furthermore, microalgae are natural producers of interesting biomolecules for commercial applications. Due to their high lipid content, they are promising sources for biofuels to replace fossil fuels. However, the commercial production of microalgal biofuels is currently not feasible due to the high costs for algae cultivation, harvesting and biomass processing (Odjadjare et al. 2017). For this reason, the generation of alternative valuable compounds by microalgae, such as food and feed additives, pigments, cosmetics and pharmaceuticals is gaining increased attention (Borowitzka 2013).

In order to further enhance natural productivities, metabolic engineering has been successfully applied to some microalgae. For example, in *Chlamydomonas reinhardtii*, the content of two carotenoids (violaxanthin and lutein) was doubled by heterologous expression of an additional phytoene synthase gene from strong endogenous promoters (Cordero et al. 2011). Similarly, in *Dunaliella salina*, a 3-fold higher accumulation of violaxanthin and zeaxanthin was achieved by heterologous expression of a β-carotene hydroxylase from *C. reinhardtii* (Simon et al. 2016). However, molecular genetic tools were restricted to the model alga *C. reinhardtii* for a long time, and only recently has sequencing of numerous algal genomes facilitated the genetic engineering of non-model algae, such as *Nannochloropsis* species (for an overview, see Poliner et al. 2018a).

*Nannochloropsis* belong to the class of *Eustigmatophyceae* within the diverse group of stramenopiles that evolved by secondary endosymbiosis of a red alga with a heterotrophic eukaryote (Qiu et al. 2013). The unicellular and coccoid *Nannochloropsis* species produce large amounts of lipids accumulating up to 60% of their biomass dry weight under stress conditions (Rodolfi et al. 2009). Additionally, they contain an extra-ordinarily high content of the health-beneficial polyunsaturated fatty acid (PUFA) eicosapentaenoic acid (up to 4.3% of biomass dry weight; Camacho-Rodríguez et al. 2014). Recently, several novel promoters for genetic engineering of *N. gaditana* have been identified (Ramarajan et al. 2019) and a number of plasmids for nuclear transformation of *N. oceanica* have become available that even enable multigene expression (Poliner et al. 2017; Zienkiewicz et al. 2017). Elevated levels of total fatty acids and PUFAs were achieved by over-expressing an endogenous diacylglycerol acyltransferase and several fatty acid desaturases (Poliner et al. 2017; Zienkiewicz et al. 2017).

In addition to metabolic engineering, microalgae can serve as hosts for recombinant protein production (Akbari et al. 2014; Rasala and Mayfield 2015). In comparison with prokaryotic expression systems, eukaryotic microalgae offer several advantages for eukaryotic proteins, such as correct folding and diverse posttranslational modifications including glycosylation. Compared with other eukaryotic expression systems like plants or insects, growth rates of microalgae are generally higher and upscaling procedures are typically easier and cheaper (Yan et al. 2016). Different antibodies and vaccines have been produced in *C. reinhardtii* upon nuclear or chloroplast transformation, with yields ranging from 0.1 to 21% of total soluble protein (TSP) (for an overview see Rasala and Mayfield 2015). *D. salina* was successfully engineered by nuclear transformation to produce a viral envelope protein for shrimp vaccination (Feng et al. 2014). A human monoclonal antibody could be produced at 8.7% of TSP by nuclear expression in the diatom *Phaeodactylum tricornutum* (Hempel et al. 2011). Moreover, microalgae elegantly allow the simultaneous production of recombinant proteins next to other high-value products like PUFAs and pigments, predestining the organisms for biorefinery approaches (Hariskos and Posten 2014). Indeed, high PUFA accumulation was recently achieved next to recombinant phytase production in *P. tricornutum* to improve phosphorus bioavailability of the algal biomass in animal feed (Pudney et al. 2019).

Several technical advances laid the foundation for possibly establishing *N. oceanica* CCMP1779 as a novel host for recombinant protein production in the future, including (i) stable transformation by random integration of expression cassettes into the nuclear genome (Vieler et al. 2012), (ii) its well-annotated genome sequence (Vieler et al. 2012; Wang et al. 2014) and (iii) multiple expression vectors (Poliner et al. 2017; Zienkiewicz et al. 2017), in combination with its high growth rate and robust and easy cultivability. Important prerequisites for successful and economically viable recombinant protein production are high expression rates and low rates of protein turnover without negatively affecting growth and photosynthesis. For gene expression, the promoter generally is the most decisive element next to introns and the terminator. Additionally, the translation efficiency may be enhanced by an N-terminal extension of the recombinant protein by a short peptide stemming from a highly abundant endogenous protein, as shown for tobacco chloroplasts (e.g. 14 aa of the ribulose bisphosphate carboxylase large chain protein, Kuroda and Maliga 2001). Improved protein yields by the addition of comparable leader sequences (LS) have also been observed in prokaryotes (e.g. *E. coli* and cyanobacteria, Betterle and Melis 2018; Sprengart et al. 1996) and upon chloroplast expression in *C. reinhardtii* (Richter et al. 2018).

In this study, we compared the strength of six *N*. *oceanica* promoters and their corresponding LS in *Venus* expression at three major levels (transcription, fluorescence and protein yield) by complementary methodology, combining the analysis of transformant populations with that of individuals. The strongest promoters allowed stable recombinant protein production up to 5% of TSP. We demonstrate that *N. oceanica* is most suitable for stable and high-yield recombinant protein production under both constitutive and auto-inducible cultivation conditions.

## Materials and methods

### Microalgal strains and cultivation

*Nannochloropsis oceanica* strain CCMP1779 was received from the National Centre for Marine Algae and Microbiota (NCMA, East Boothbay, USA) and cultivated in f/2 medium prepared with 3.3% (w/v) artificial sea water (ASW, Tropic Marin, Wertenberg, Germany) according to Guillard and Ryther (1962). Compared with standard growth medium used for 48- and 96-well plates and 100-ml batch cultures (1.8 mM NaNO_3_ and 72 μM NaH_2_PO_4_), 5-fold elevated nitrate and phosphate concentrations were provided in photobioreactors (PBR, 400-ml batch cultures, 9 mM NaNO_3_ and 0.36 mM NaH_2_PO_4_). Solidified f/2 medium contained 1.5% (w/v) agar and only half ASW concentration (1.65% w/v). The light regime was 16/8-h light/dark at 22 °C ± 1. The light intensity was 75 μmol m^−2^ s^−1^ for small-scale liquid cultivation (48- and 96-well plates, 100-ml batch cultures) and agar plates and 110 μmol m^−2^ s^−1^ for PBRs. PBR cultures were constantly stirred in 500-ml bottles and supplied with a CO_2_/air mixture (1% (v/v) CO_2_, flow rate 1 l/min) during the light period (Gris et al. 2013). To compensate for medium acidification by CO_2_ bubbling, the pH of the f/2 was initially adjusted to pH 9.2 and dropped to pH 7.8 after 2 h of bubbling. The first main culture was inoculated with a 100-ml batch pre-culture grown to logarithmic phase (OD_540_ = 0.8–1.0; OD_540_ = 1.0 is equivalent to 2.1*10^7^ cells/ml) to a starting OD of 0.02. After 6 days, typically an OD = 1.5 was reached and used for inoculation of the second main culture (starting OD = 0.5). For the development of the auto-induction medium for NR promoter transformants, the cells were pre-grown in 2 mM NH_4_Cl (first main PBR culture) and shifted to 10 mM nitrate with 0, 0.4 or 0.8 mM NH_4_Cl (second main PBR culture).

### Construction of transformation vectors

**Fig. 1:**
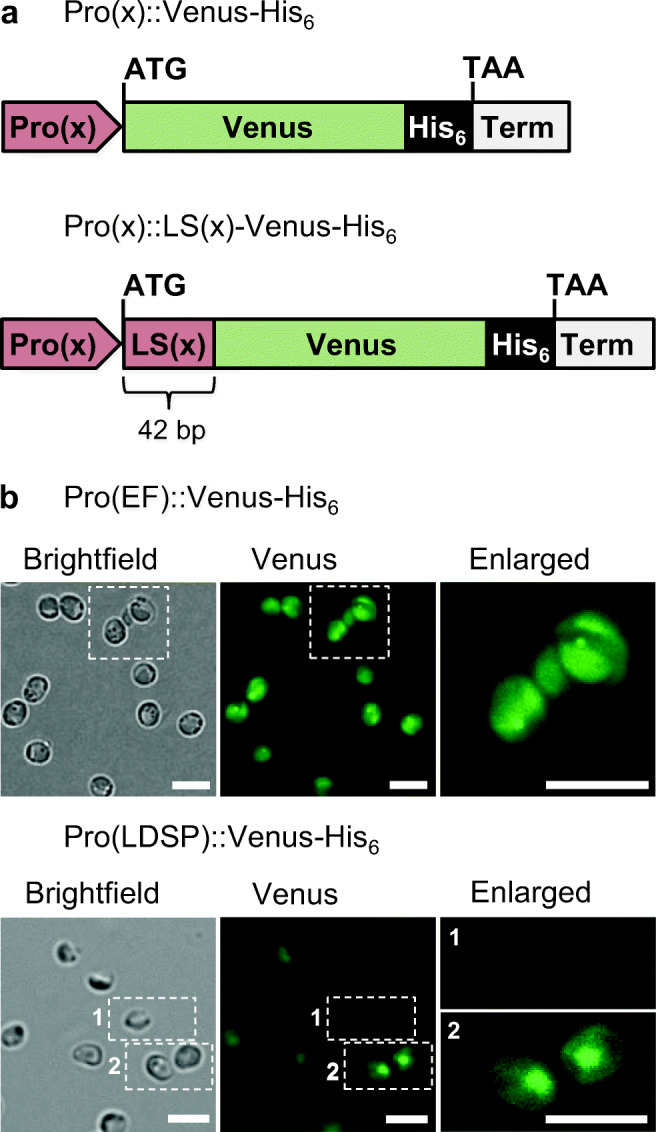
Schematic diagram of two types of expression cassettes used for promoter strength analyses and microscopic images of stable *N. oceanica* transformants. **a** Different endogenous *N. oceanica* promoters were cloned either with or without the first 42 bp of their CDS to drive expression of the reporter gene *Venus*, which was extended by a C-terminal hexahistidine tag for Venus quantification by immunoblotting. Five different promoters were tested (Pro(x), x = elongation factor, lipid droplet surface protein, nitrate reductase, α-tubulin and violaxanthin/ chlorophyll a binding protein). All expression cassettes contained the terminator of lipid droplet surface protein. **b** Exemplary and representative microscopic images of the progeny of two stable *N. oceanica* transformants, showing either strong and relatively homogenous *Venus* expression (EF promoter) or weak and heterogeneous yellow fluorescence (LDSP promoter). The two magnified image sections show one non-fluorescent cell with minimal auto-fluorescence (1) and one cell with moderate Venus fluorescence (2). EF, elongation factor; LDSP, lipid droplet surface protein; LS, leader sequence; Pro, promoter; Term, terminator. Scale bar: 5 μm

As subcloning basis to generate stable *N. oceanica* CCMP1779 transformants, the expression vector pNoc ox Venus was used (Zienkiewicz et al. 2017). This vector possesses one expression cassette consisting of the promoter of the lipid droplet surface protein (LDSP), the *HygR* gene for resistance to hygromycin B and the nopaline synthase terminator and a second cassette for the expression of the reporter gene controlled by the elongation factor (EF) promoter and terminated by the LDSP terminator. As a reporter gene, we chose *mVenus*, a monomeric GFP derivative. Subcloning was performed according to standard protocols (Sambrook and Russell 2001) using enzymes from ThermoScientific™ (Waltham, USA). For C-terminal His_6_-tag addition, the *Venus* gene was amplified from of the original plasmid with a forward primer containing a *Mun*I restriction site and a reverse primer adding the His_6_-tag and a *Mlu*I site (Table S1) to replace the untagged *Venus* gene. To generate the two different construct types for each promoter with and without leader sequence (LS, (Pro(x)::Venus and Pro(x)::LS(x)-Venus, Fig. 1a), the original EF promoter was removed by *Not*I and *Mun*I and replaced by different endogenous promoters (Table S2), either alone or extended by the corresponding 42-bp LS, as predicted by the *N. oceanica* genome (Vieler et al. 2012; https://mycocosm.jgi.doe.gov/Nanoce1779_2/Nanoce1779_2.home.html), and was amplified from genomic DNA (Table S1). Genomic DNA of *N. oceanica* was isolated using Cetyltrimethylammonium bromide (Varela-Alvarez et al. 2006). To ensure comparability of the EF promoter construct without and with LS, a 24-bp non-coding region located between the 3′-end of the promoter sequence and the start codon of *Venus* in the original pNoc ox Venus vector was removed (Table S1).

### Transformation of *N. oceanica* by electroporation

*N. oceanica* was grown under standard conditions to mid-exponential growth phase and transformed by electroporation with 3 μg vector DNA (linearized by *Ahd*I or *Psi*I) and 30 μg salmon sperm DNA (Vieler et al. 2012). Single colonies were grown on selective plates containing 50 μg/ml hygromycin, were transferred to 96-well plates containing 200 μl f/2 medium with hygromycin and were incubated for 7–10 days under standard growth conditions.

### Confocal microscopy

For imaging, a Leica DMi8 inverted microscope coupled to the confocal spinning disc unit CSU X1 (Yokogawa Electric Corporation, Musashino, Japan) was used. The system was equipped with a 515-nm laser for excitation of Venus. Images were acquired by VisiView software (Visitron Systems, Puchheim, Germany). Confocal images were captured as single planes with a sCMOS camera system (QImaging OptiMOS).

### Flow cytometric analysis

**Fig. 2:**
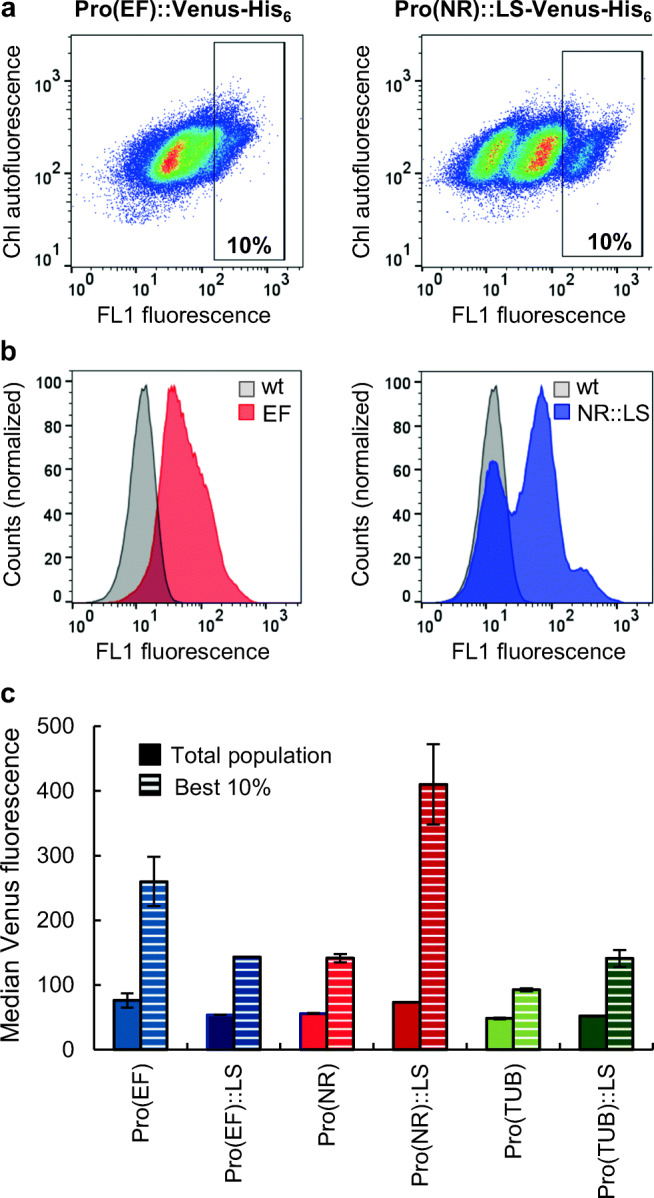
Comparative flow cytometry analyses of Venus fluorescence and chlorophyll autofluorescence for selected populations of *N. oceanica* transformants. **a** Density diagrams of chlorophyll (Chl) autofluorescence (FL2-Area) against FL1 fluorescence (FL1-Area), visualizing the heterogeneity of transformants in Venus fluorescence, which spanned two orders of magnitude for the EF promoter (w/o LS, left) and the NR::LS promoter (right). **b** Histogram of Venus fluorescence distribution in the two transformant populations compared with the low autofluorescence of the wild type (wt) detected by the FL1 channel. Contrary to the single peak for the EF promoter, the transformants for NR::LS (right) formed three distinct subpopulations differing in Venus fluorescence. **c** Each Venus-positive population was selected (gated) based on the corresponding overlay diagrams of the fluorescence of the transformant population and the autofluorescence of the wild type (Fig. S2). To quantify population-specific differences in Venus fluorescence, median Venus fluorescence values were calculated either for the entire population of Venus-positive transformants or for the best 10% of the highest Venus fluorescence (boxed in **a**). The standard deviation of two biological replicates is given. The corresponding density diagrams and histograms of the remaining four construct types (EF::LS, NR, TUB±LS) are shown in Suppl. Fig. S1

Venus fluorescence was analysed in transformant populations by growing at least 40 different *N. oceanica* transformants of each promoter construct individually in 1 ml in 48-well plates for 5 days under standard growth conditions until mid-exponential growth phase. Cells were pooled in equal proportions based on cell number and subjected to flow cytometric analysis using the S3e cell sorter (Bio-Rad, Hercules, USA) equipped with a 488-nm excitation laser. To allow comparability between experiments, the flow cytometer was calibrated using proline universal and rainbow calibration beads (Bio-Rad, Hercules, USA) according to the manufacturer’s instructions. *N. oceanica* cells were diluted to approx. 10^6^ cells/ml and analysed at a rate of 500 events/s (10^5^ cells in total for each sample). The data was collected using the instrument specific acquisition software. To determine the median Venus fluorescence, the data was analysed by the FlowJo software package (v10.6.1; BD Life Science, Ashlan, USA) with the following gating strategy: *N. oceanica* cells were first identified based on their morphological features in a two-dimensional density plot of side scatter area (SSC-Area) versus forward scatter area (FSC-Area). Chlorophyll-positive cells were identified in a density plot of FL2-Area (560-nm long pass filter) versus FSC-Area and displayed in a density plot diagram of FL2-Area versus FL1-Area (525/30-nm bandpass filter) or in an FL1-Area histogram to visualize the Venus fluorescence of the transformant population in comparison with the background fluorescence of wild-type cells (Figs. 2, S1 and S2). The median Venus fluorescence was calculated after gating for the Venus-positive population.

### *Venus* expression analysis by qPCR

At least 40 individual *N. oceanica* transformants were separately grown in 48-well plates for 5 days, as described above, pooled, harvested by centrifugation (15 min, 4000*g*) and frozen in liquid nitrogen. RNA was isolated from these cells, as described by Vieler et al. (2012), using Trizol™ (Invitrogen™, USA) and the RNeasy® Mini Kit with DNaseI digest (Qiagen, Hilden, Germany). After a second DNaseI digest (Thermo Scientfic™, USA), total cDNA was generated from 200 ng RNA using the RevertAid First Strand cDNA Synthesis kit (Thermo Scientific™, USA) and an oligo(dT)_18_ primer. Relative *Venus* expression analyses of the pooled transformants were carried out with the primer pairs given in Table S1 and with *actin2* (*ACT2*) as a reference gene (transcript ID: 6413). The qPCR was performed with the Green Master Mix (Roche, Mannheim, Germany) and 1.5 μl of cDNA as template using the LightCycler® 96 System (Roche, Germany). The primer efficiencies were determined at least for five 10-fold cDNA dilutions (10^−1^ to 10^−6^ ng/μl) for *Venus* (99%) and *ACT2* (92%). Normalized *Venus* expression (as fold change) was calculated by the ΔΔCt method (Pfaffl 2001), using *Venus* expression from the TUB promoter population as calibrator.

### Quantification of Venus fluorescence by plate reader measurement

Venus fluorescence of alive *N. oceanica* transformants was quantified in triplicates using a Synergy HT microplate reader (Biotek Instruments, Vermont, USA) in flat clear bottom black 96-well microplates (BRANDplates®, Wertheim, Germany). Venus fluorescence was measured in 100 μl adjusted to 7.5*10^6^ cells/ml against f/2 medium using a 485/20-nm excitation and a 528/15-nm emission filter. For calculation of the so-called cellular Venus fluorescence, the absolute fluorescence values were normalized to OD_750_.

### Protein isolation and immunoblotting

For protein isolation, approx. 10^8^ cells of *N. oceanica* cultured in PBRs were sedimented (3500 g for 15 min) and resuspended in lysis buffer (50 mM Tris/HCl, pH 8.0, 1 mM EDTA and 2x Roche cOmplete, EDTA-free protease inhibitor) according to Chu et al. (2016). The cells were lysed using glass beads (0.5 g, diameter 0.5 mm) by four to five cycles of shaking (6500 rpm for 30 s) in a Precellys homogenizer (Bertin instruments, Montigny-le-Bretonneux, France) with sample cooling on ice to avoid sample overheating and protein degradation. Cell debris was removed by centrifugation and the supernatant frozen in liquid nitrogen. The concentration of total soluble protein (TSP) was quantified in the supernatant (Bradford 1976). For immunoblotting, proteins were separated by SDS-PAGE and transferred electrophoretically onto nitrocellulose membrane (BioTrace NT; Pall Life Sciences, Portsmouth, USA) using a Criterion™ Blotter (Bio-Rad). Venus was detected with a primary rabbit anti-GFP antibody (dilution 1:5.000, ab290; abcam, Berlin, Germany) and a secondary horseradish peroxidase-coupled antibody. For absolute quantification of Venus-His_6_ in TSP, a standard curve was generated using a purified His_6_-tagged calibration protein (approx. 14 kDa; dynamic range between 0.05 and 0.8 μg; Fig. S5). The latter and Venus-His_6_ were detected by a primary mouse anti-His_6_ antibody (1:10,000 dilution, ab18184; abcam) and the corresponding peroxidase-coupled secondary antibody. Peroxidase was detected by enhanced chemiluminescence (Amersham ECL Prime Western Blotting Detection Reagent; GE Healthcare, Chicago, USA) and a Fujifilm imager with a CCD camera (LAS-3000; Fujifilm, Tokyo, Japan). Images were analysed by the ImageJ 1.521 software package (Schneider et al. 2012).

## Results

### Analysis of endogenous *N. oceanica* promoters by fluorescence microscopy

To establish *N. oceanica* as a host for recombinant protein production, we selected six endogenous promoters (Table S2). As constitutive promoters, the genes coding for elongation factor (EF), α-tubulin (TUB), lipid droplet surface protein (LDSP) and two violaxanthin chlorophyll a binding proteins (VCP-1/-L) were chosen. As predicted inducible promoter, that of nitrate reductase (NR) was selected. Each promoter sequence was tested with and without the addition of a leader sequence (LS) composed of the first 42 bp (i.e. 14 aa) of its own ORF fused N-terminally to the reporter protein (Fig. 1a).

For the generation of stable *N. oceanica* CCMP1779 transformants, we selected a suitable backbone vector of the pNoc ox series containing two separate expression cassettes; one for the expression of the hygromycin resistance gene and the other for the reporter gene of interest (Zienkiewicz et al. 2017). The constitutive EF promoter was already available for reporter gene expression and was extended by its LS. The five additional promoter sequences (±LS) were amplified from genomic DNA of *N. oceanica* with appropriate restriction sites to replace the original EF promoter. As a reporter gene, we chose a cytosolic version of monomeric *Venus*, which is a yellow fluorescent GFP derivative with advantageous properties in terms of increased brightness and maturation speed, abolished dimerization tendency, and reduced detection interference with chlorophyll autofluorescence (Nagai et al. 2002).

*N. oceanica* CCMP1779 was transformed with the newly created expression vectors by electroporation with a typical transformation rate of approx. 70 hygromycin-resistant colonies per microgram of plasmid DNA. The transformants were selected first on agar plates and subsequently propagated in 96-well plates (Vieler et al. 2012). First, the signal strength, stability and homogeneity of Venus fluorescence were examined in clonal transformant populations by confocal spinning disk microscopy (Table S2). Strong and evenly distributed cytosolic Venus fluorescence was observed in specific transformants of three promoters (EF, NR and TUB, each ±LS), as shown representatively for the EF promoter (Fig. 1b). However, different transformants of the same promoter constructs showed remarkable variations in Venus fluorescence (data not shown). In contrast, uniformly low fluorescence was detected in all transformants of the two LDSP and all four VCP promoter constructs (Fig. 1b, data not shown). Hence, subsequent analyses focused on the EF, NR and TUB promoter transformants (±LS) with high promoter activity.

### Promoter strength analysis by flow cytometry in transformant populations

Due to the variations in Venus fluorescence among individual transformants of the same promoter constructs (see above), we next applied flow cytometry to quantify the distribution of the Venus fluorescence in transformant populations using the Bio-Rad S3e cell sorter. Forty individual transformants of the same construct type were mixed in equal proportions by cell number to one transformant population, which allowed averaging of reporter gene expression independent of the transformants’ growth rates. As an indicator for cell viability, chlorophyll-positive cells were detected with the FL2 channel and Venus fluorescence was detected with the FL1 channel (Fig. 2a). Chlorophyll autofluorescence of wild-type *N. oceanica* was only weakly detected by the FL1 channel (Figs. S1 and S2). For each promoter construct, a Venus positive sub-population was clearly distinguishable from the autofluorescence of the wild-type population (Figs. 2b and S1). After gating for Venus-positive cells, three transformant populations (EF and TUB±LS) formed one large Venus-specific unimodal population that spanned up to two orders of magnitude (i.e. 100-fold difference in fluorescence). These three populations and that of the NR promoter had the lowest median fluorescence values among the six Venus positive population, ranging from 48 to 56, while higher median values were obtained for the EF and NR::LS populations (76 and 73, respectively). Interestingly, the latter two transformant populations showed a multimodal distribution with sub-populations of very high Venus fluorescence, demonstrating a high heterogeneity.

Fluorescence-activated cell sorters not only allow high-sensitivity analyses of microalgae but also sorting of single cells with superior properties. We next calculated the median Venus fluorescence for those 10% of transformants with the highest Venus fluorescence. As hypothesized, the differences among the promoter constructs became more pronounced (Fig. 2c). The median Venus fluorescence values of both the EF promoter (260) and the NR::LS promoter population (410) exceeded that of the TUB promoter population 2.8- and 4.5-fold, respectively (Fig. 2c). Moreover, for all three promoters, the Venus fluorescence was affected by the LS. Atypically, the median Venus fluorescence from the EF promoter population lacking the LS was > 1.8-fold higher compared with that of the EF::LS construct. In contrast, for the TUB and NR promoter population, the LS addition had a positive enhancing effect, resulting in a 1.5-fold increase in Venus fluorescence for the TUB promoter and even in a nearly 3-fold increase in case of the NR promoter (Fig. 2c).

### Quantitative promoter strength analysis in transformant populations by qRT-PCR

**Fig. 3:**
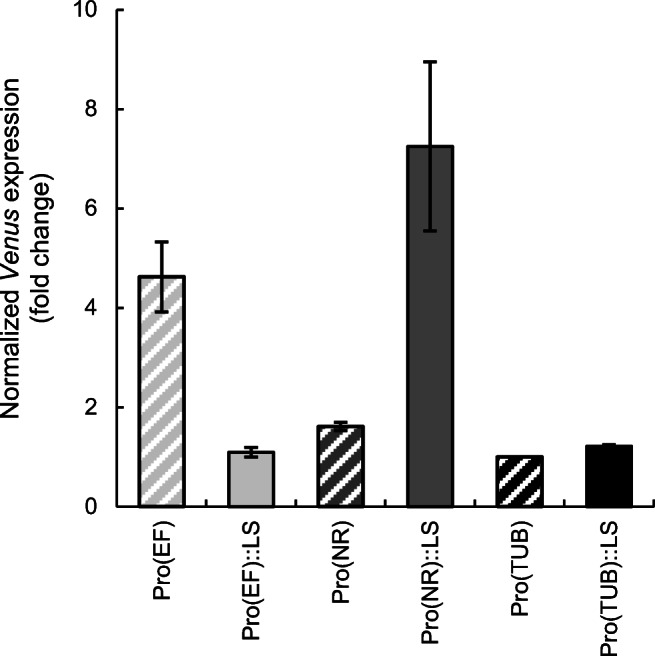
Comparative qPCR analyses of *Venus* expression in populations of *N. oceanica* transformants. *Venus* expression was determined in populations of least 40 individual transformants by qPCR, using *ACT2* as the reference. *Venus* expression of the TUB promoter population (Pro(TUB)::Venus) was used as the calibrator. Mean values of normalized *Venus* expression with SD of two biological replicates are shown

In order to compare the strength of different promoters at the RNA level and independent of any fluorophore (e.g. for untagged recombinant proteins), we established gene expression analysis by qPCR for transformant populations. In this approach, the same populations analysed by flow cytometry (see above) were subjected to expression analysis (Table S2). *Venus* expression was normalized to expression of the housekeeping reference gene *ACT2* (Cao et al. 2012) and *Venus* expression from the TUB promoter was used as calibrator applying the ΔΔCt method (Pfaffl 2001). *Venus* expression of the transformant populations of EF::LS and TUB::LS was rather similar to that of the TUB population, while *Venus* expression from the NR promoter was 1.6-fold higher. The highest *Venus* transcript expression was determined for the EF and NR::LS promoter transformants, namely 5- and 7.5-fold higher, respectively, relative to the TUB promoter transformants (Fig. 3). Overall, the results of the expression analysis were largely consistent with the flow cytometry data. Notably, the differences in *Venus* expression between the pooled EF and NR promoter transformants compared with their LS containing variants were more pronounced, as compared with the flow cytometry results (Fig. 2c).

### Growth phase-dependent Venus productivity analyses

**Fig. 4:**
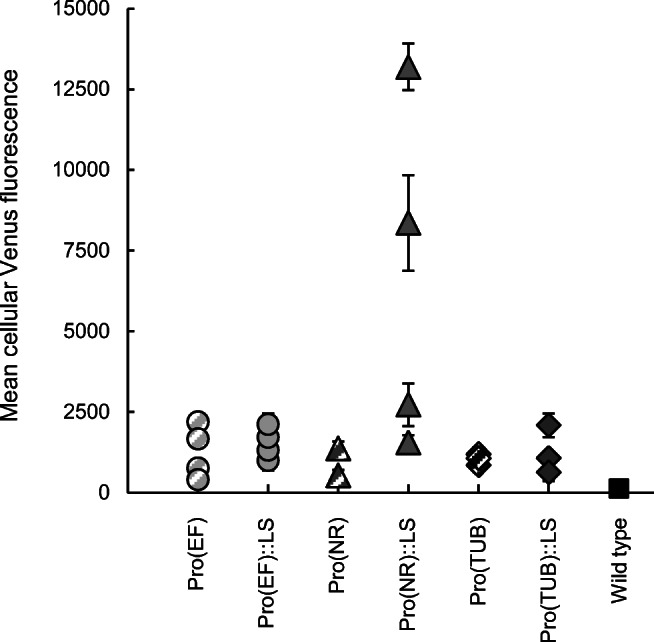
Comparison of the mean cellular Venus fluorescence of *N. oceanica* transformants. For each construct, at least three different transformants were grown in 100-ml batch cultures and the “cellular Venus fluorescence” (fluorescence/OD) was quantified daily over the growth period (Fig. S3) by fluorescence plate reader measurements. In the exponential growth phase, cellular Venus fluorescence was rather constant, as shown representatively for two constructs (Pro(EF)±LS, Fig. S3), and was averaged to the mean cellular Venus fluorescence (and SD), shown here for each transformant. Two NR::LS promoter transformants had an extra-ordinarily high mean cellular fluorescence of 8400 and 13,200. As negative control, the autofluorescence of wild-type cells was quantified similarly by the same filter sets and was rather low (133 and 172, two biological replicates)

For recombinant protein production, individual transformants with high promoter activity are required that allow high and stable protein production over the entire growth period and which are not compromised in growth rate. For this reason, we quantified the Venus fluorescence (normalized to OD_750_; referred to as cellular Venus fluorescence) over the entire growth period in batch cultures using a plate reader equipped with suitable filters. The filters detected only low levels of chlorophyll autofluorescence for wild-type *N. oceanica* (Fig. 4). For each promoter construct, at least three individually grown transformants were analysed. In the EF promoter transformants (±LS), the cellular Venus fluorescence was stable in the exponential growth phase (OD_540_ 0.5 to 2.0, Fig. S3), while it started declining slightly earlier in the NR promoter transformants (OD_540_ = 1.5, data not shown). The TUB promoter transformants showed low and instable cellular fluorescence (data not shown). For each transformant analysed, those cellular Venus fluorescence values that were rather constant during the growth period were averaged (Fig. S3). This so-called mean cellular Venus fluorescence value was the highest for several individual transformants of both EF promoter constructs (±LS) and the LS containing NR promoter. Interestingly, two NR::LS promoter transformants showed a more than 5-fold higher mean cellular Venus fluorescence compared with the transformants lacking this N-terminal 14-aa peptide (Fig. 4).

### Determination of Venus protein yields in small-scale photobioreactors

**Fig. 5:**
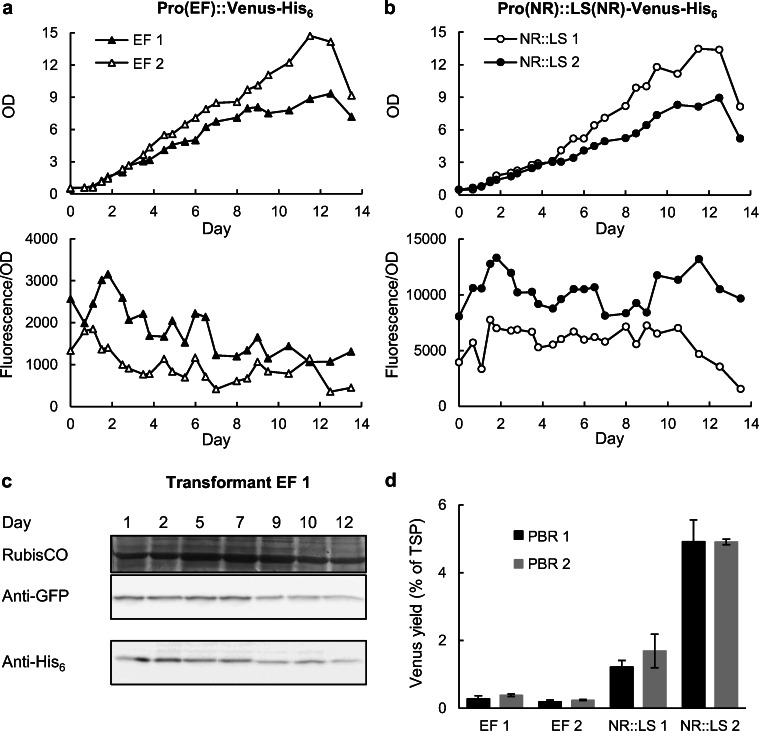
Analysis of cellular Venus fluorescence, protein production and protein yields of individual *N. oceanica* transformants under constitutive growth conditions in PBRs. **a** For two transformants of the EF promoter, the cellular fluorescence (fluorescence/OD) was analysed over the growth period. One of two PBR replicates is shown. **b** Similar analyses were carried out for two transformants of the NR::LS promoter. **c** Kinetic analysis of Venus protein content in cellular extracts of total soluble protein (TSP), shown representatively for EF 1, by immunoblotting with anti-GFP (5 μg TSP) and anti-His_6_ specific antibodies (40 μg TSP). Both antibodies did not cross-react with any protein of approx. 27 kDa when loading TSP of wild-type *N. oceanica* as negative control (shown in Fig. S4a). The Coomassie stained large subunit of RubisCO (51 kDa) served as loading control. Venus degradation was undetectable and the cross-reactivity of both antibodies correlated well (see also Fig. S4b). **d** In the late exponential growth phase (typically day 9), Venus yields were quantified by anti-His_6_ immunoblotting (in % of TSP), using a purified His_6_ protein for calibration (Fig. S5). For each transformant, the Venus yields of two independent PBR experiments were analysed (PBR1 and 2), and for each of them, the mean value of two technical replicates is given. Details of the quantification method are provided in Fig. S6

For high yields, stable recombinant protein accumulation up to high cell densities is an important prerequisite. Based on the previous results (Fig. 4), two EF promoter transformants (referred to as EF 1 and EF 2) and two NR::LS promoter transformants (NR::LS 1 and NR::LS 2) were chosen for Venus productivity analyses in small-scale photobioreactors (PBR). The transformants were cultivated under constant supply with CO_2_/air (1%/99% v/v) during the light period in 400-ml PBRs. Cellular Venus fluorescence was analysed over the growth period until stationary phase (Fig. 5a and b). In general, the growth rate of different transformants was similar (0.20 to 0.25 day^−1^), but they differed in maximum OD in stationary phase (9 to 14). Typically, cellular Venus fluorescence increased shortly after inoculation of the main culture, indicating that Venus biosynthesis was higher than the “dilution effect” by cell division in the lag phase (Fig. 5a and b). In exponential growth phase, cellular fluorescence decreased slightly and remained relatively constant, before the values declined in the stationary growth phase. Interestingly, one NR::LS promoter transformant, NR::LS 2, showed an extra-ordinarily high cellular Venus fluorescence oscillating around 11,000 in exponential growth phase (Fig. 5b), which was approximately 8- to 10-fold higher than the best EF promoter transformant (EF 2; Fig. 5a).

PBR cultivation allowed sufficient production of biomass and protein (typically up to 150 μg of total soluble protein (TSP) from 8*10^8^ cells) to analyse the accumulation of Venus-His_6_ protein by immunoblotting with a primary anti-GFP-specific antibody at several time points over the whole growth period. Accordingly, Venus accumulation was stable without protein degradation until late exponential growth phase (Fig. 5c). Additionally, we established relative determination of recombinant protein yield using a primary anti-His_6_-specific antibody as a fluorophore independent quantification method for future applications. For this, an unrelated purified His_6_-tagged protein was selected as a calibration standard (Fig. S5). The GFP- and His_6_-specific immunoblotting data correlated very well and showed roughly the same trend as the semi-quantitative data of the cellular Venus fluorescence (Figs. 5c; S4b). Venus yields per TSP were quantified in cells harvested in the late exponential growth phase (typically day 9) when the cellular fluorescence was still stable. Two best EF promoter transformants, EF 1 and EF 2, produced moderate Venus yields of 0.2%–0.3% of TSP. By contrast, remarkably high Venus yields of 1.5% (NR::LS 1) and 4.9% of TSP (NR::LS 2) were achieved for the best NR::LS promoter transformants. Hence, approx. 0.42 mg recombinant Venus protein could be produced by a 400-ml PBR within 9 days.

### Development of an auto-induction system based on ammonium repression and nitrate inducibility of the NR promoter

**Fig. 6:**
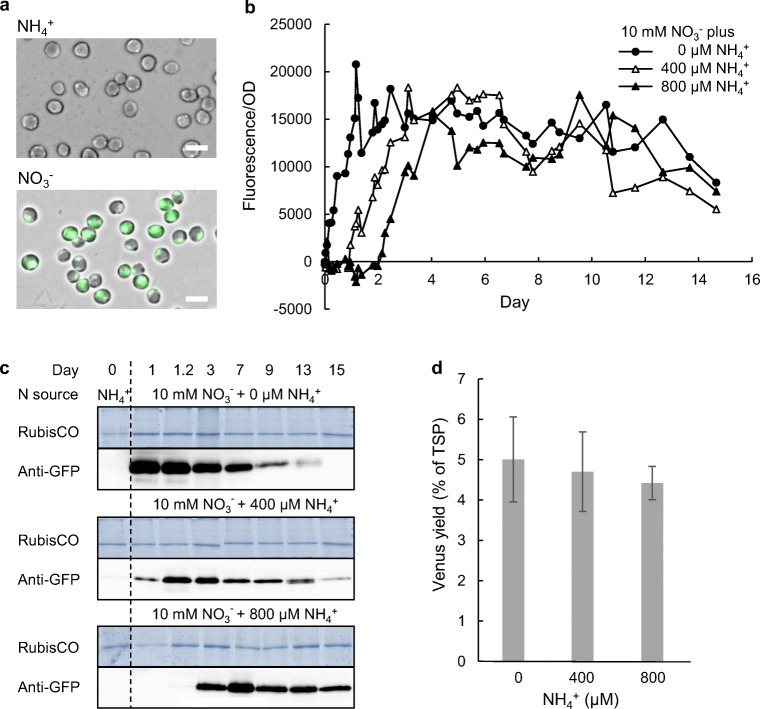
Inducible, N source-dependent Venus protein accumulation upon expression from the NR promoter. **a** Microscopic analysis of Venus fluorescence (green, shown as overlay with brightfield image) in NR promoter transformants confirmed that Venus expression was repressed by ammonium (NH_4_^+^) and induced by nitrate (NO_3_^−^). Scale bar 5 μm. **b** To develop an auto-induction medium, the best NR::LS transformant was precultured in 2 mM ammonium and subsequently shifted to 10 mM nitrate either alone, or with 400 or 800 μM ammonium. Cellular Venus fluorescence was measured over the growth period, as representatively shown for one of two parallel PBR experiments. Increasing ammonium concentrations indeed delayed the induction of Venus fluorescence. **c** Analysis of Venus protein content by anti-GFP immunoblotting (1 μg of TSP per lane) confirmed a correlation between cellular fluorescence (**b**) and Venus protein (27 kDa). RubisCO LSU served as a loading control. **d** Venus yields were quantified by anti-His_6_ immunoblotting (Fig. S5b) on day 9 and averaged for two biological replicates, each analysed in two technical replicates (5 μg of TSP). Details are shown in Fig. S7b

Up to this point, the NR promoter transformants were exclusively grown under constitutive growth conditions using solely nitrate as the N source. Though nitrate is the common N source in f/2 medium, *N. oceanica* prefers the fully reduced ammonium, which can be directly incorporated into α-ketoglutarate and glutamate for amino acid biosynthesis. In plants and several algae, the nitrate reductase promoter is repressed by ammonium and induced by nitrate (Berges 1997; Solomonson and Barber 1990). To investigate whether the NR promoter of *N. oceanica* had the same properties, we grew the corresponding transformants first in ammonium and then switched the growth medium to nitrate. Indeed, cellular Venus fluorescence remained undetectable in the presence of ammonium by fluorescence microscopy but became clearly visible upon switching to nitrate (Fig. 6a). Hence, the NR promoter of *N. oceanica* is fully repressed by ammonium and induced by nitrate.

To develop an auto-induction medium for recombinant protein production from the NR promoter in *N. oceanica*, we investigated whether low ammonium concentrations in the presence of high nitrate concentrations could (i) repress *Venus* expression in the lag and very early exponential growth phase to generate moderate cell concentrations; (ii) slowly induce *Venus* expression and (iii) enable the same high levels of Venus fluorescence and protein yields at the harvest time point in the early stationary phase. Indeed, a low ammonium concentration of 400 μM (next to 10 mM nitrate) delayed *Venus* induction by approximately 24 h and high cellular fluorescence (> 9000) was detected at OD 1.5 (Figs. 6b and S7a). By doubling the ammonium concentration to 800 μM, cellular Venus fluorescence became detectable after > 50 h and reached the same high cellular fluorescence at moderate cell densities (> 9000 at OD 3.0; Figs. 6b; S7). The cellular fluorescence correlated well with immunoblotting results, since a strong Venus-specific cross-reaction could only be detected after 1 day (for 400 μM NH_4_^+^) and 3 days (for 800 μM NH_4_^+^; Fig. 6c). Notably, independent of the initial repressive effect of ammonium, similarly high levels of cellular Venus fluorescence were reached for all three culture conditions, oscillating around 12,000 to 13,000 (Fig. 6b). Quantification by immunoblotting revealed that the maximum Venus yields of 5.1% of TSP (0 μM NH_4_^+^) were only slightly reduced in the presence of ammonium (400 μM: 4.4% of TSP; 800 μM: 4.1% of TSP; Fig. 6d). In summary, these ammonium and nitrate concentrations can serve as auto-inducing conditions for recombinant protein production to shift the induction time point from lag phase to exponential growth phase when cultures reach moderate cell densities without compromising in final protein yields. Such an auto-induction medium is particularly important for future applications in producing toxic recombinant proteins.

## Discussion

*Nannochloropsis* species are evolutionarily highly divergent from each other and from related families. Consequently, heterologous gene expression generally requires the use of endogenous promoters (Akbari et al. 2014; Schroda et al. 2000). To develop an expression system for economically viable, high-yield recombinant protein production in *N. oceanica*, we investigated the expression strength of six different endogenous promoters with and without the corresponding LS by analysing and quantifying *Venus* expression, fluorescence and protein yields by a set of complementary methodologies. The original EF promoter of the basal pNoc ox vector had previously been primarily used for metabolic engineering of fatty acid metabolism by (over-)expressing a diacylglycerol acyltransferase and several fatty acid desaturases (Poliner et al. 2017; Zienkiewicz et al. 2017). Our newly cloned promoter sequences stemmed from five different genes involved in diverse physiological functions of the cell. TUB proteins are the major constituent of microtubuli building the cytoskeleton and the respective TUB promoter was able to drive constitutive gene expression in *N. oceanica* IMET1 (Li et al. 2014; Wang et al. 2016). The two VCP promoters had, for example, been used in the first published CRISPR/Cas9 system for *N. oceanica* (Wang et al. 2016). The LDSP promoter successfully expressed the hygromycin resistance gene in the pNoc ox vectors (Zienkiewicz et al. 2017). The promoter of the *NR* gene is nitrate-inducible in higher plants and had so far been studied in *C. reinhardtii* (Loppes et al. 1999), *Chlorella vulgaris* (Niu et al. 2012), *P. tricornutum* (Chu et al. 2016) and *N. gaditana* (Jackson et al. 2018).

In this study, the choice of a fluorescent reporter gene proved extremely useful since it enabled (semi-) quantitative protein analyses by fluorescence microscopy, plate reader and flow cytometry. Transformant analysis by fluorescence microscopy allowed efficient pre-screening of transformants. High levels of cytosolic Venus fluorescence were detected for three promoters (EF, TUB and NR) under constitutive growth conditions. In contrast, the six other promoter variants (LDSP and VCP-1/-L, ±LS) generated only low Venus fluorescence close to the detection limit and were considered unsuitable for substantial recombinant protein production. Additionally, we detected significant differences in *Venus* expression, not only between promoter constructs but also among individual transformants of the same construct. Such heterogeneity in gene expression strength in transformant populations is typical for nuclear transformation, random genome integration and single or multiple cassette insertion, contrary to single site-specific integration by homologous recombination (Ramarajan et al. 2019; Schroda 2019).

Large differences in gene expression between individual transformants make it challenging to determine the expression strength of different promoters in a comparable and reproducible manner. Quantitative RT-PCR and flow cytometry of transformant populations, as established here, proved ideal in analysing and averaging *Venus* expression at the mRNA and protein level, respectively. The EF and NR::LS populations showed the highest *Venus* expression with 5- and 7.5-fold higher *Venus* expression, respectively, by qPCR compared with the LS lacking TUB population. Expression analysis and fluorescence data determined by flow cytometry showed the same trend for the different promoter constructs, in particular when the median fluorescence value was calculated for the best 10% of the cells with the highest fluorescence. This value was 3-fold (EF population) and 4.5-fold (NR::LS) higher than the fluorescence of the respective TUB population (Figs. 2c and 3). Collectively, flow cytometry is a valuable tool for quantitative promoter strength comparison in reporter gene expression and to determine enhancing effects of genetic elements. While flow cytometry relies on fluorophore tags, qPCR is more flexible and fluorophore independent.

We further investigated if the extension of the promoters by the first 42 bp of the corresponding CDS enhanced Venus expression, as reported for *E. coli* (Sprengart et al. 1996), *Synechocystis* (Betterle and Melis 2018), *C. reinhardtii* (Richter et al. 2018) and tobacco chloroplasts (Kuroda and Maliga 2001; Ye et al. 2001). For example, the addition of an N-terminal 14-amino acid LS boosted protein yield in tobacco chloroplasts by a factor of 30 (Ye et al. 2001). In our experimental system, we consider it unlikely that the small 42-bp addition of the LS influenced the site or the number of expression cassette insertions into the nuclear genome. We concluded that some but not all LS indeed enhanced gene expression. This enhancer effect was most pronounced for the NR promotor (4.5-fold higher *Venus* expression and nearly 3-fold increased fluorescence of the 10% best transformants). However, two other LS (of EF and TUB gene) did not or only marginally enhanced *Venus* expression. Hence, the LS effect was construct-specific in this study, as reported previously (Richter et al. 2018), and remains largely unpredictable. In general, the translation-enhancing effect of specific LS, also called downstream boxes, is not yet understood mechanistically in detail. To the best of our knowledge, translation-enhancing effects of LS have only been described for prokaryotic or plastid-based expression systems. Our enhancer effect of the LS of NR on *Venus* gene expression is thus the first documented result for a nuclear-encoded eukaryotic gene. Future studies need to address to what extent the LS of NR also enhances translation of recombinant genes other than *Venus*. It will be interesting to combine the strong constitutive EF promoter with the LS of NR to possibly further increase the strength of that expression cassette.

In exponential and early stationary growth phase, recombinant protein biosynthesis per cell is generally constant. Thus, maximum microalgal productivity per day is reached at high cell densities. As a drawback, the rate of recombinant protein production and protein quality usually decreases towards the late exponential phase due to insufficient light penetration, a lack of nutrients (N and P) and microelements and due to elevated endogenous proteolytic activity. We therefore performed in-depth analyses of Venus fluorescence over the entire growth period with focus on the late exponential to early stationary phase (Fig. 5). Venus accumulation was stable over the entire growth period without any detectable protein degradation according to anti-GFP and anti-His_6_ immunoblot analyses (Figs. 5C). Similar to qRT-PCR, immunoblotting based on the His_6_ epitope tag as the antigen is applicable to any non-fluorescent recombinant protein of interest in future studies.

Our Venus yields achieved under constitutive cultivation conditions in PBRs ranged from 0.2 to 0.3% of TSP for the EF promoter strains, which is in the range of < 1% typically reported in algae for both nuclear and plastidic expression. For instance, the V28 protein as subunit vaccine against the white spot syndrome virus was produced by nuclear expression in *Dunaliella salina* with a yield of 0.3% of TSP (Feng et al. 2014), and a recombinantly produced Hepatitis B surface antigen reached a yield of 0.7% of TSP in *P. tricornutum* (Hempel et al. 2011). Most importantly, our Venus yields achieved with the NR::LS promoter protein under both constitutive and inducible growth conditions were extraordinarily high (i.e. 1.5 to 4.9% of TSP). Previously, such high yields had been hardly reported for *Nannochloropsis*. Only based on a theoretical protein content per cell, a similarly high GFP yield of 1.5 ± 1.1% of TSP had been calculated for the NR promoter in *N. gaditana* (Jackson et al. 2018). Interestingly, a recombinantly produced small antimicrobial peptide fused to red fluorescent protein accumulated to up to 4.3% of TSP in the cytosol of *N. salina,* when using a combined promoter of the heat shock protein 70A and RubisCO SSU from *C. reinhardtii* (Li and Tsai 2009). Similar high recombinant protein levels had been typically achieved only by chloroplast transformation of *C. reinhardtii*. For example, a yield of 3% of TSP was achieved for a fusion vaccine composed of a subunit of the foot-and-mouth disease virus and cholera B toxin (Sun et al. 2003). Heterologous expression of a bioactive mammalian protein even reached 5% of TSP (Manuell et al. 2007). Only in very few cases even higher yields have been reported like for the V28 protein produced in the *C. reinhardtii* plastid with a yield of > 20% of TSP (Surzycki et al. 2009).

Although protein production in chloroplasts is generally advantageous in terms of protein yields and stability, we here show that high yields of intact protein can also be produced upon nuclear transformation. In contrast to plastid expression, nuclear transformation does not require the time-consuming generation of homoplasmic lines. Hence, newly generated nuclear transformants are directly available for upscaling procedures and analytics. Notably, our *N. oceanica* transformants remained stable even without selection pressure (unpubl. data), as reported for *N. gaditana* (Jackson et al. 2018), which reduces the costs for industrial-scale cultivation.

The production of toxic proteins negatively impacts cell viability and proliferation. For this reason, auto-induction media are applied to induce gene expression at moderate cell densities. The high strength of the NR promoter combined with its nitrate inducibility and repression by ammonium (Fig. 6a) opened the possibility to develop an inducible expression system for *N. oceanica*. The rapid nitrate-dependent induction of the NR promoter and its repression by ammonium (Fig. 6a and b) had previously been described for *P. tricornutum* (Chu et al. 2016) and in parallel studies for *N. gaditana* (Jackson et al. 2018) and *N. oceanica* (Poliner et al. 2020). Notably and contrary to our results, the NR promoter of *N. gaditana* retained residual and non-negligible activity in the presence of both ammonium and nitrate (Jackson et al. 2018), preventing its application in an auto-induction system. Moderate concentrations of ammonium (up to 800 μM) fully repressed *Venus* expression for approx. 2 days (Fig. 6b and c). After consumption of ammonium as the preferred N source, *Venus* expression was quickly induced by 10 mM nitrate at elevated OD_540_ (3.0), while maintaining the final high Venus yield at culture harvest. Depending on the degree of recombinant protein toxicity in future projects, the auto-induction conditions may be fine-tuned. Hence, we here describe a novel ammonium/nitrate-based auto-induction system for recombinant protein production from the NR promoter. This is the first auto-induction system for *Nannochloropsis* developed for diverse time- and cost-efficient biotechnological applications in the near future.

The strength of the identified strong EF and NR promoters needs to be verified in combination with different genes of interest since it is commonly known that promoter strength may vary dependent on the coding sequence (Schroda 2019). Additionally, the effect of terminator sequences on gene expression levels and protein yields is often neglected and offers further optimization potential. For example, in *C. reinhardtii*, the same promoter combined with different terminators showed up to 40-fold differences in gene expression (Kumar et al. 2013). Quantitative analyses by flow cytometry established in this study can be applied straightforward to further optimize promoter/terminator variations*.*

In the past few years, many research groups made major technological contributions to genetic engineering of *Nannochloropsis* (and particularly *N. oceanica*), including gene stacking methods using novel bidirectional promoters and viral P2A and marker-free gene editing tools (Poliner et al. 2017, 2018b, 2020). An elegant combination of the technological advancements with our results will ultimately enable the establishment of *N. oceanica* as a host for recombinant protein production. Potential economic applications are diverse and unlimited. For instance, *N. oceanica* may be used to produce animal vaccines to orally immunize fish against widespread diseases in aquaculture (Charoonnart et al. 2018).

## Electronic supplementary material

ESM 1 (PDF 606 kb)

## Data Availability

The authors will make scientific material available upon request.
