# Supplementary material for: Development of a constitutive and an auto-inducible high-yield expression system for recombinant protein production in the microalga Nannochloropsis oceanica
Source: Appl Microbiol Biotechnol. 2020 Sep 9;104(20):8747–60. doi: 10.1007/s00253-020-10789-4 (PMC7502441; doi:10.1007/s00253-020-10789-4)
Supplement: Supplementary file 1 — (PDF 606 kb) [file 253_2020_10789_MOESM1_ESM.pdf]

"Development of a constitutive and an auto-inducible high-yield expression system for recombinant protein production in the microalga *Nannochloropsis oceanica*"

Journal: *Applied Microbiology and Biotechnology*;

Authors: Imke de Grahl, Sweta Suman Rout, Jodi Maple-Grødem and Sigrun Reumann;
Affiliation: Plant Biochemistry and Infection Biology, Institute of Plant Science and Microbiology, Universität Hamburg, D-22609 Hamburg, Germany;
Email address of corresponding author: sigrun.reumann@uni-hamburg.de

Supplementary Material

Figure S1

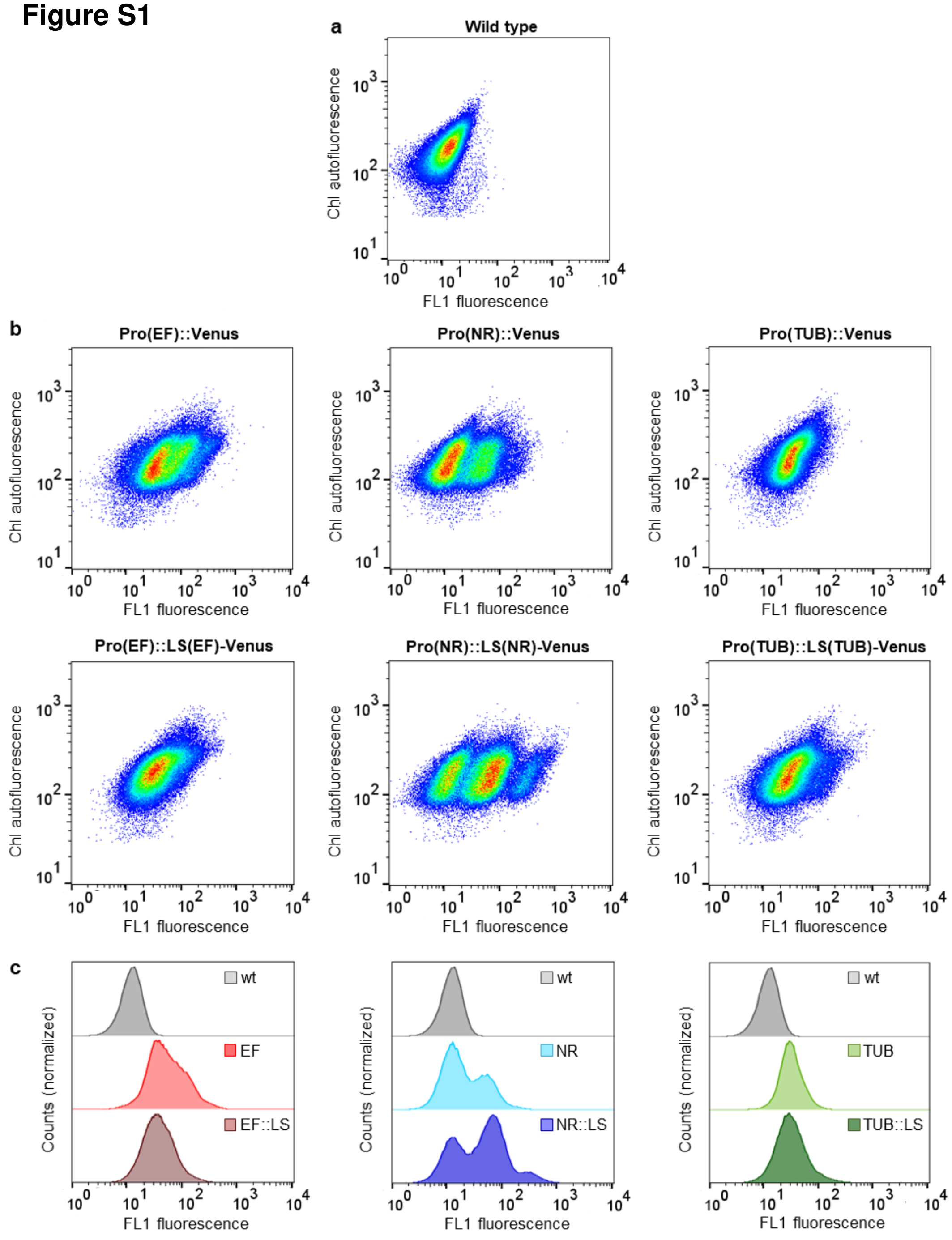

Fig. S1: Density diagrams and histograms of comparative flow cytometry analyses of Venus fluorescence and chlorophyll autofluorescence of *N. oceanica* transformants. a As negative control, wild-type (wt) *N. oceanica* cells were analyzed in a density diagram for chlorophyll (chl) autofluorescence (FL2-Area, y axis) against fluorescence detected by the FL1 channel (FL1-Area, x axis). For wild-type cells, weak chl autofluorescence was detected by the FL1 channel. **b** In six different transformant populations of the EF, NR and TUB promoter constructs (±LS), the corresponding density diagrams show high Venus fluorescence that exceeds the low chl autofluoresence, both detected with the FL1 channel. **c** Histogram of Venus fluorescence distribution in the same six transformant populations, showing the low autofluoresence of wild type (grey) in comparison to the Venus fluorescence of the transformant populations.

Figure S2

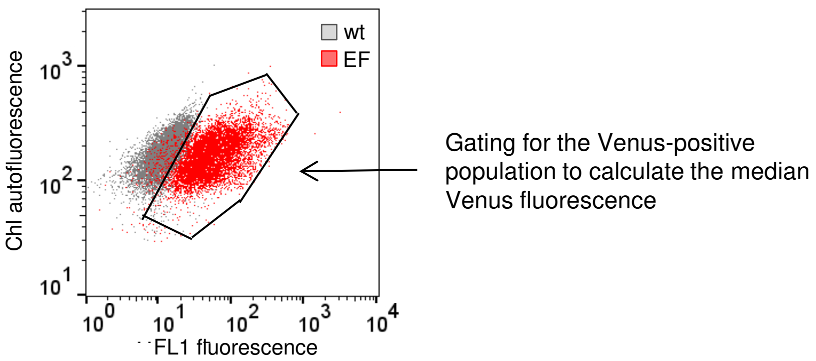

Fig. S2: Overlay density diagrams of FL1 fluorescence and chlorophyll autofluorescence of *N. oceanica* wild-type cells and one *Venus* expressing promoter population (EF). For calculation of the median Venus fluorescence the Venus positive population was selected from an overlay density diagram of the transformant population (here Pro(EF); red) and the wild type (wt, grey). The region marked with black line was used to calculate the median Venus fluorescence.

Figure S3

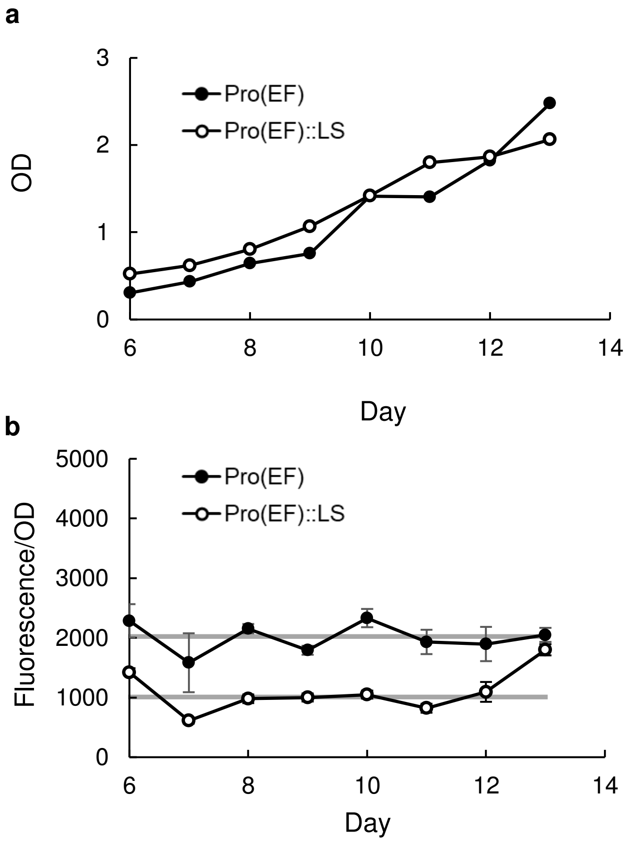

Fig. S3: Determination of mean cellular Venus fluorescence of individual transformants. Two representative EF promoter transformants (±LS) were analysed for growth characteristics (a) and cellular Venus fluorescence (fluorescence/OD, b). For each time point, the fluorescence/OD of three technical replicates (±SD) is given. During the exponential growth phase (OD 0.5 to 2.0), cellular Venus fluorescence was relatively stable. The mean cellular Venus fluorescence (given as grey line) was calculated as the mean value of fluorescence/OD between OD 0.5 to 2.0 (Pro(EF): 2000±300; Pro(EF)::LS: 1000±300).

Figure S4

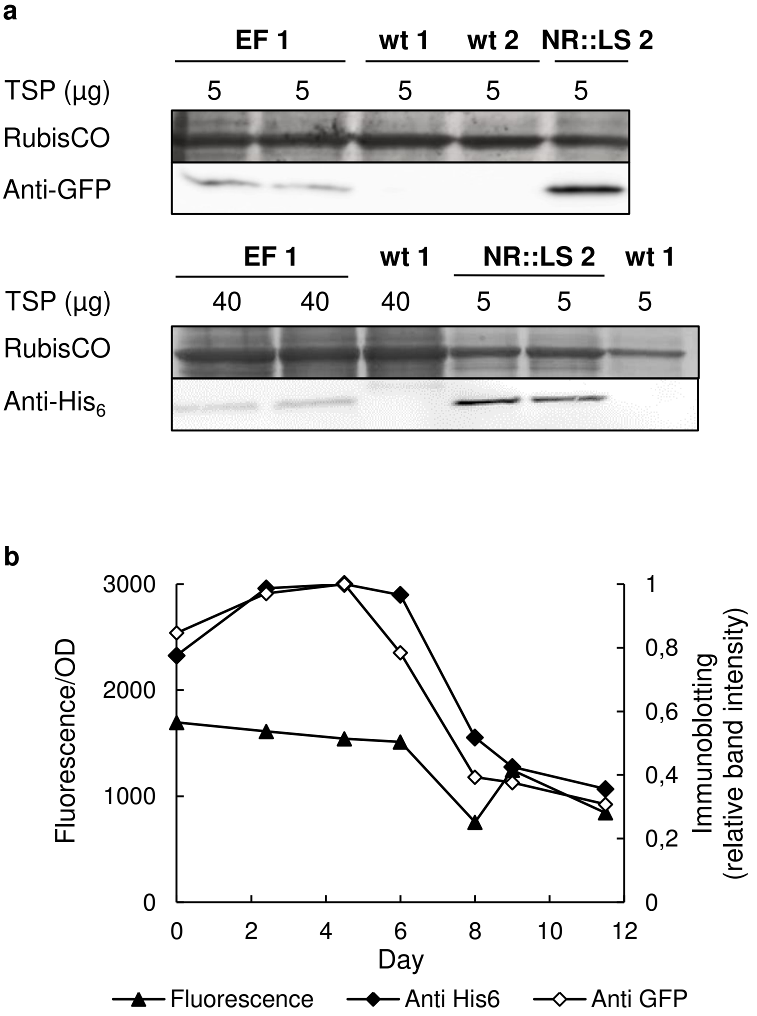

Fig. S4: Negative immunoblotting controls for wild-type *N. oceanica* (wt) and correlation between cellular Venus fluorescence and relative Venus-His$_6$ protein content. a Detection of Venus-His$_6$ in total soluble protein (TSP) extracts from two specific transformants (EF 1 and NR::LS 2) by immunoblotting with anti-GFP and anti-His$_6$ specific antibodies. As negative control, no cross-reacting protein of approx. 27 kDa was detected for the wildtype (replicates wt 1 and wt 2). The Coomassie stained large subunit of RubisCO (51 kDa) served as loading control. The loaded amounts of TSP are indicated. **b** For one transformant (EF 1), the relative levels of recombinant Venus-His$_6$ were determined with anti-GFP and anti-His$_6$ antibodies over the whole growth phase for 5 µg and 40 µg of TSP loaded, respectively, and compared to the fluoresence/OD.

Figure S5

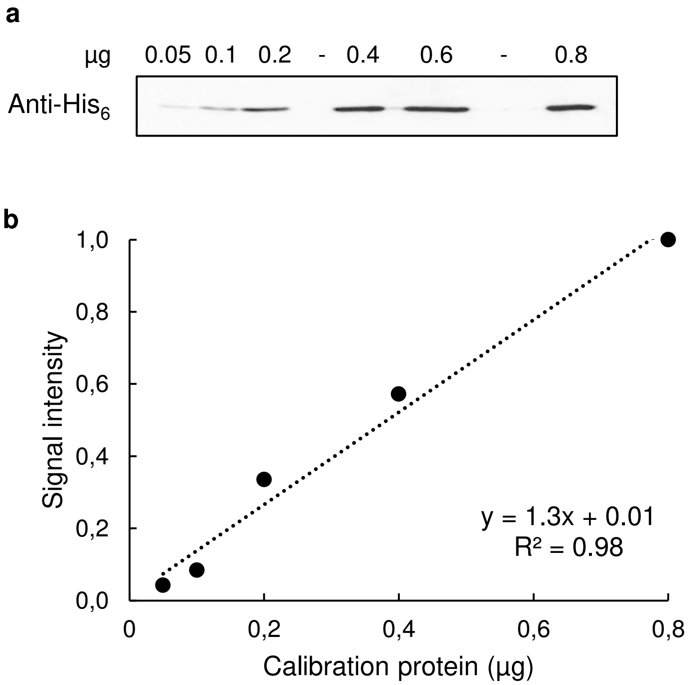

Fig. S5: Standard curve of a purified His_6-tagged calibration protein to quantify Venus yields in *N. oceanica* transformants. a Different amounts (0.05 to 0.8 µg) of the His_6-tagged calibration protein (approx. 14 kDa) were detected by immunoblotting using an anti-His_6 specific primary antibody. **b** Venus-His_6-specific band intensities were normalized to 0.8 µg of protein by ImageJ and plotted against the amount of calibrator protein to generate a standard curve. The value for 0.6 µg of calibration protein was considered an outlier and excluded from linear regression analysis.

Figure S6

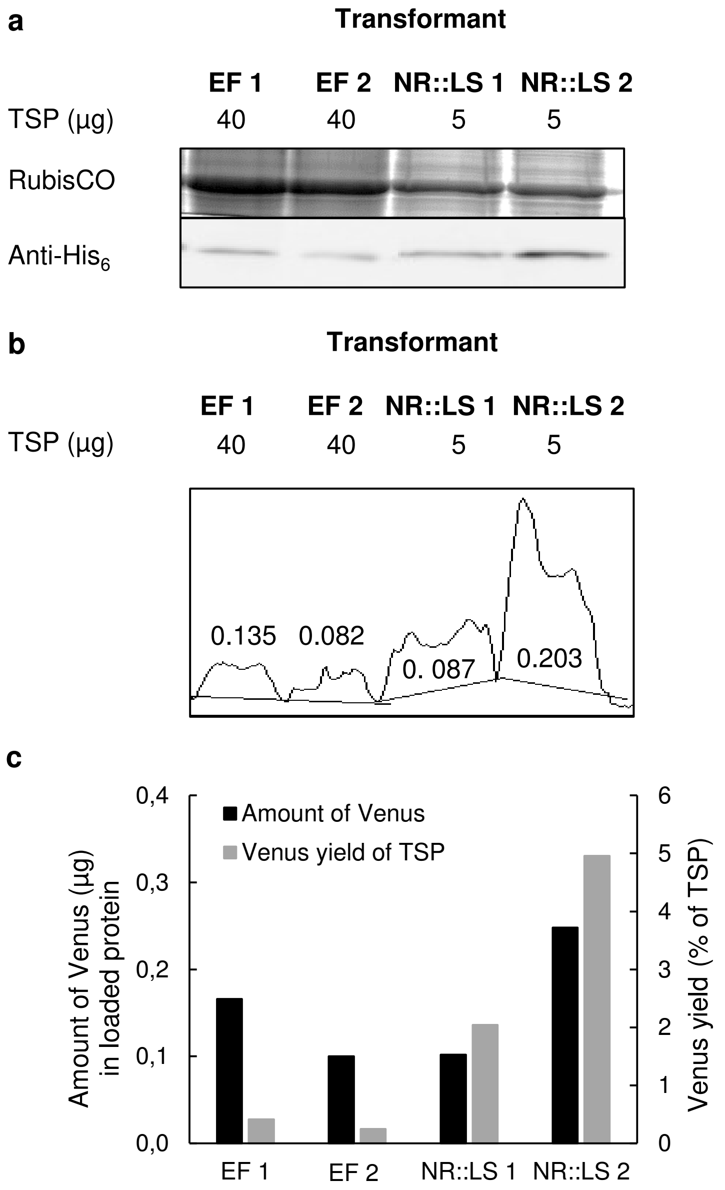

Fig. S6: Exemplary quantification of Venus yields of *N. oceanica* transformants by anti-His$_6$ specific immunoblot analysis. a Venus-His$_6$ was detected in cellular extracts of total soluble protein (TSP) of four transformants (EF 1, EF 2, NR::LS 1 and NR::LS 2) by immunoblotting with anti-His$_6$ specific antibodies. The Coomassie stained large subunit of RubisCO (51 kDa) served as loading control. The amounts of loaded TSP are indicated. **b** ImageJ quantification of each band of transformants shown in (a) is depicted as surface area peaks and the relative signal intensities areas are indicated. **c** The amount of Venus in loaded protein (in µg; primary y axis) was calculated from the relative signal intensities using the experiment specific calibration curve and standard equation (here: $y=1.65x+0.0071$) and the conversion factor of the molecular weight of the pure His$_6$-tagged protein to Venus-His$_6$ which is 2.1. The Venus yields (in % of TSP) calculated from the loaded amount of TSP are indicated on the secondary y axis.

Figure S7

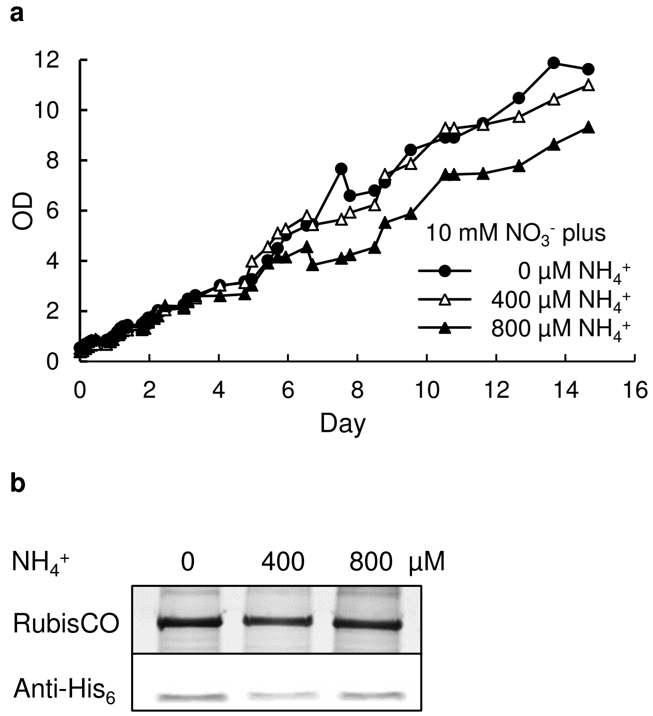

Fig. S7: Analysis of the repressive effect of different ammonium concentrations (in the presence of 10 mM nitrate) on transformant growth (a) and Venus-His$_6$ yield (b) for one representative *N. oceanica* transformant. a One transformant (NR::LS-Venus-His$_6$, NR::LS 2) was pre-grown in 2 mM ammonium (to repress Venus expression) and shifted to inducible conditions (10 mM nitrate) in the presence of no or low concentrations of ammonium (0, 400 or 800 µM) to gradually delay the induction of Venus expression to higher OD_{540} until the consumption of ammonium as the easier accessible N source. One representative growth curve of two biological replicates is shown. **b** Venus yields in TSP were analyzed after 9 days. In the ammonium containing auto-induction media, Venus yields remained high, e.g. 4.1% of TSP for 800 µM NH_4^+ (compared to 5.1% of TSP for 0 µM NH_4^+). Constant protein load of 5 µg per lane was verified by the Commassie stained large subunit of RubisCO.

Table S1: Primers used for subcloning and qRT-PCR analysis. Restriction sites are underlined.

Construct	Primer name	Primer sequence (5'-3')
**Subcloning primers**		
Venus-His$_6$	Venus His$_6$ MunI for	AACCAATTGGGGAGCGGCATGGTG
	Venus His$_6$ MluI rev	AAAACGCGTTCAGTGATGGTGATGGTGATGGCCGCTTCC TTTGTACAACTCATCCATCCCAAGCG
Pro(EF)::Venus-His$_6$	EF pro NotI fw	AAGCGGCCGCTATAGCTACATGGTAGCTAGTAG
	EF pro MunI rv	AAACAATTGTGTTACGAAGTGAGGGTTGAGGGG
Pro(EF)::LS(EF)-Venus-His$_6$	EF pro NotI fw	AAGCGGCCGCTATAGCTACATGGTAGCTAGTAG
	EF pro LS MunI rv	AAACAATTGGCCAATCACGACCAGGTTCACGTGG
Pro(NR)::Venus-His$_6$	NR pro MunI fw	AAACAATTGGTAGAGGAGCAACGTGCTAAAGC
	NR pro NotI rv	AAAGCGGCCGCATTTTTATGTCAGACGCAAGGT
Pro(NR):: LS(NR)-Venus-His$_6$	NR pro LS MunI fw	AAACAATTGCTCAGGCGCCGTAGGCACCTGTGGTGAGAG
	NR pro NotI rv	AAAGCGGCCGCATTTTTATGTCAGACGCAAGGT
Pro(TUB)::Venus-His$_6$	TUB pro MunI fw	AAACAATTGGGTTGAGACTAGTTGGAGGGAGGAG
	TUB pro NotI rv	AAAGCGGCCGCATCATATCGTGCCACAGCAGATTGG
Pro(TUB):: LS(TUB)-Venus-His$_6$	TUB pro LS MunI fw	AAACAATTGGTTTCCGCATTGCCCAGCTTGGAC
	TUB pro NotI rv	AAAGCGGCCGCATCATATCGTGCCACAGCAGATTGG
Pro(VCP1)::Venus-His$_6$	VCP1 pro NotI fw	AAAGCGGCCGCCACTGAACCTGTCCGCATCCTG
	VCP1 pro MunI rv	AAACAATTGGAGGGTGGAGACAGTGAGGAG
Pro(VCP1):: LS(VCP1)-Venus-His$_6$	VCP1 pro NotI fw	AAAGCGGCCGCCACTGAACCTGTCCGCATCCTG
	VCP1 pro LS MunI rv	AAACAATTGGGAGAACTTGGGGGCGGGGG
Pro(VCP-L)::Venus-His$_6$	VCP-L pro NotI fw	AAAGCGGCCGCCTTGCCTATCTTGTCTTAGTGCCG
	VCP-L pro MunI rv	AAACAATTGACTTAAGAAGTGGTGGTGGTGGTG
Pro(VCP-L):: LS(VCP-L)-Venus-His$_6$	VCP-L pro NotI fw	AAAGCGGCCGCCTTGCCTATCTTGTCTTAGTGCCG
	VCP-L pro LS MunI rv	AAACAATTGGCCCATAAGGGTGGAGACAGTGAGGAG
Pro(LDSP)::Venus-His$_6$	LDSP pro NotI fw	AAGCGGCCGCATGGAGGAGGAGGCGAGCGTAGC
	LDSP pro MunI rv	AAACAATTGTGTTGATGCGGGCTGAGATTGGTG
Pro(LDSP):: LS(LDSP)-Venus-His$_6$	LDSP pro NotI fw	AAGCGGCCGCATGGAGGAGGAGGCGAGCGTAGC
	LDSP pro LS MunI rv	AAACAATTGCGTGGTCGCGGAGGGCGCAGAGG
**qPCR Primers**		
ACT2 (Transcript-ID: 6413)	ACT2 for	ACCTTCTACAACGAGCTGC
	ACT2 rev	GAACGTCTCAAACATAATCTGG
Venus	Venus for	GCCGAAGTGAAGTTTGAGG
	Venus rev	GTCCGCGGTGATATACACG

Table S2: List of endogenous *N. oceanica* promoters analysed in this study. Protein and transcript IDs of the respective promoters are given according to the nomenclature by the Joint Genome Institute (JGI, https://mycocosm.jgi.doe.gov/Nanoce1779_2/Nanoce1779_2.home.html). The length of the cloned promoters is provided. The methods of transformant analyses are specified for each promoter construct. Individual transformants were analysed by fluorescence microscopy, fluorescence quantification (plate reader) and immunoblotting (Venus protein), while transformant population were quantitatively analysed by flow cytometry and qRT-PCR. For the TUB promoter transformants, Venus was only quantified by fluorescence rather than additionally by immunoblotting (*). TF, transformant, n.d., not determined.

**Abbreviation**	**Gene name**	**Protein (transcript ID)**	**Cloned promoter length (bp)**	**Method of transformant analysis**		
				Fluorescence microscopy	Flow cytometry and qPCR	Fluorescence quantification and immunoblotting
				Individual TF	TF populations	Individual TF
EF	Elongation factor	544243 (544329)	991	yes	yes	yes
LDSP	Lipid droplet surface protein	591558 (591644)	744	yes	n.d.	n.d.
NR	Nitrate reductase	590448 (590534)	794	yes	yes	yes
TUB	α-tubulin	592433 (592519)	562	yes	yes	yes*
VCP1	Violaxanthin/ chlorophyll a binding protein 1	603160 (603246)	863	yes	n.d.	n.d.
VCP-L	Violaxanthin/ chlorophyll a binding like protein	589384 (589470)	585	yes	n.d.	n.d.
